# Variation of adverse drug events in different settings in Africa: a systematic review

**DOI:** 10.1186/s40001-024-01934-0

**Published:** 2024-06-16

**Authors:** Linda Nyame, Yuhua Hu, Hui Xue, Emmanuel D. K. Fiagbey, Xi Li, Yong Tian, Lijun Fan, Wei Du

**Affiliations:** 1 School of Public Health, Southeast University, Nanjing, China; 2 School of Basic Medicine and Clinical Pharmacy, China Pharmaceutical University, Nanjing, China; 3 Health Research Institute, Faculty of Health, University of Canberra, Bruce, ACT Australia

**Keywords:** Adverse drug events, Adverse drug reactions, Africa, Prevalence, Systematic review

## Abstract

**Background:**

Adverse drug events (ADEs) represent challenges affecting Africa’s healthcare systems owing to the increased healthcare expenditure and negative health outcomes of ADEs.

**Objectives:**

We aimed to systematically review published studies on ADEs and synthesize the existing evidence of ADE prevalence in Africa.

**Methods:**

Studies reporting on ADE occurrence in African settings and published from Jan 1, 2000 to Oct 1, 2023 were identified by searching PubMed, EBSCO, Science Direct, and Web of Science. Studies that either articulately investigated ADEs caused by clinical condition (such as HIV patients) or ADEs caused by exposure to specific drug(s) (such as antibiotics) were considered specific and the remaining were general. Grouped ADE prevalence rates were described using median and interquartile range (IQR). PROSPERO registration (CRD42022374095).

**Results:**

We included 78 observational studies from 15 African countries that investigated the prevalence of ADEs leading to hospital admissions (17 studies), developed during hospitalizations (30 studies), and captured in the outpatient departments (38 studies) or communities (4 studies). Twelve studies included multiple settings. The median prevalence of ADE during hospitalization was 7.8% (IQR: 4.2–21.4%) and 74.2% (IQR: 54.1–90.7%) in general and specific patients, respectively. The ADE-related fatality rate was 0.1% and 1.3% in general and specific patients. The overall median prevalence of ADEs leading to hospital admissions was 6.0% (IQR: 1.5–9.0%); in general, patients and the median prevalence of ADEs in the outpatient and community settings were 22.9% (IQR: 14.6–56.1%) and 32.6% (IQR: 26.0–41.3%), respectively, with a median of 43.5% (IQR: 16.3–59.0%) and 12.4% (IQR: 7.1–28.1%) of ADEs being preventable in general and specific patients, respectively.

**Conclusions:**

The prevalence of ADEs was significant in both hospital and community settings in Africa. A high ADE prevalence was observed in specific patients, emphasizing important areas for improvement, particularly in at-risk patient groups (e.g., pediatrics, HIV, and TB patients) in various settings. Due to limited studies conducted in the community setting, future research in this setting is encouraged.

**Supplementary Information:**

The online version contains supplementary material available at 10.1186/s40001-024-01934-0.

## Introduction

Patient safety ranks among the most paramount priorities of healthcare professionals. Nevertheless, there are still substantial challenges to assure patients their safety allowing for the increasing complexities of healthcare delivery [1, 2]. Despite advances in healthcare, ADEs result in an upsurge in morbidity and mortality [3], such as extended hospital stays, and increased healthcare expenditures [4], particularly for the elderly and children [5, 6], thereby exerting significant burden on patients' recovery and healthcare systems.

According to a 2018 systematic review, including 18 hospital-based studies, ADEs significantly caused morbidities in African hospitals, with 8.4% inpatients encountering ADEs during hospitalization and 2.8% owing to ADEs causing hospital admissions, of which 43.5% of these ADEs were assessed preventable [7]. The prevention of ADEs in Africa can be achieved when patients who receive drugs are continuously monitored through collaborative efforts among healthcare teams, medication therapy management, and competent pharmacovigilance systems, which are the main bones in ensuring patient safety [8, 9]. Assessing the occurrence of ADEs in the outpatient or community settings to complement evidence in the hospital settings may help prioritize healthcare areas that need improvement, which in turn could assist in targeted quality improvement programs. There is a sparse amount of evidence offering a comprehensive assessment of the burden of ADEs occurring in the outpatient or community settings in Africa. There have been two systematic reviews carried out in Africa [7, 10], but these reviews had a focus on hospital settings without inclusion of community settings and adverse reactions induced by specific pharmaceutical interventions (e.g., antiretroviral therapy). The exclusion of outpatient and community settings suggest that previous studies provided an incomprehensive insight into the true burden of ADRs in Africa. This, limited insight, presents a drawback in making comprehensive policy and informed decisions to implement effective strategies for improved medication safety in Africa.

This systematic review aimed to comprehensively characterize the prevalence, preventability, seriousness or severity, and mortality of ADEs occurring in the inpatient, outpatient, and community settings in Africa, with such evidence to inform better clinical decision-making to enhance the safer use of medicines.

## Methods

### Terminology

Adverse drug events (ADEs) are unintentional injuries resulting from exposure to a medication that encapsulates adverse events [11]. Adverse drug reactions are noxious and undesired responses to medications at normal doses [12]. Study populations included in this review were grouped into two different types of population groups, i.e., general patient groups (i.e., ADEs occurring in all patients treated within a certain healthcare setting) and specific patient groups (i.e., ADEs caused by clinical condition (such as HIV patients) or ADEs caused by exposure to specific drug(s) (such as antibiotics). Community settings referred to care delivered outside a hospital inpatient or a clinic setting. Outpatient settings referred to care delivered within healthcare facilities catering to a wide range of non-urgent medical conditions. Prevalence was defined as the proportion of patients with ADE(s) within a defined patient group. Serious ADEs referred to those resulted in hospital admissions or prolonged stay during an existing hospitalization, persistent or significant disability or incapacity, or even death [13], while severe ADEs included those that were potentially life-threatening, prolonged hospitalization and caused permanent disability or fatal. Preventable ADEs referred to primarily those caused by medication errors that could generally be avoided.

### Search strategy and data source

We report the findings of this review according to the Preferred Reporting Items for Systematic Reviews and Meta-Analyses guidelines [14]. The study protocol was registered with PROSPERO (CRD42022374095). Our search began on Jan 1, 2022, and on Oct 15, 2023, a new search was conducted for updates. Studies published from Jan 1, 2000 to Oct 1, 2023 were identified by searching four electronic databases: PubMed, EBSCO, Science Direct, and Web of Science. The search query was a combination of Boolean Operators (AND and OR) and terms related to ADEs, settings of interest, and African countries. A detailed search strategy is provided in Additional file 1. In addition, we hand searched the references list of included studies and citations to retrieve relevant studies. Endnote X7 was used to organize the final search results (Thomson Reuters, Times Square New York, NY, USA) and duplicates were removed.

### Eligibility criteria

The inclusion criteria comprised of the following, i.e., observational studies that investigated the prevalence [proportion of patients with ADE(s) within a defined patient group] and/or nature (e.g., preventability and fatality) of ADEs in any patient population of all ages who were hospitalized as a result of ADEs, had developed ADE during hospitalization, or experienced ADE in the outpatient or community settings, including retrospective, prospective and cross-sectional studies; studies on adverse events were only included if injuries caused by drugs were recorded; studies conducted in an African setting; and studies that either specifically investigated ADEs associated with clinical condition(s) or exposure to specific drug(s). There was no age or disease limit in the current study selection. Studies were excluded based on the following criteria, i.e., Non-English language and non-African settings; lack of full-text availability; conference abstracts, case control studies, commentaries, or reviews; knowledge and attitudes about ADE reporting.

### Selection process

The article titles and abstracts were independently screened by two reviewers (LN and YH) to determine the relevance to the inclusion and exclusion criteria. After initial screening, the full-text of potentially relevant papers identified were further screened by two reviewers (LN and YH). The eligibility criteria outlined above were used to determine the full-text retrieval analysis of studies. When necessary, two reviewers were engaged for additional clarity on any points of contention. Differences in opinions was resolved by a third reviewer (HX).

### Data extraction

Two reviewers independently extracted data from the included studies and entered them into a data-collecting form. Study characteristics, e.g., author name, year, country, data source and duration, study design, study setting, ADE definition, population characteristics, identification of ADEs in terms of detection method, causality, seriousness or severity, fatality, preventability of ADEs, and prevalence of ADEs, were extracted from the selected studies.

### Quality assessment

Two reviewers assessed the methodological quality of included observational studies using the ten-question criteria proposed by Smyth et al. [15], considering that the widely used risk assessment approaches for systematic review such as Cochrane Risk of Bias tool and GRADE were primarily appropriate for randomized controlled trials. This ten-point quality assessment instrument employed in the current review was developed specifically for studies investigating ADEs in clinical settings and has been applied by previous systematic reviews [7, 10, 16, 17]. We replaced the term severity with “seriousness or severity” in Smyth et al.’s. assessment criteria [15]. Any remaining inconsistencies were referred to and resolved by a third reviewer. The following characteristics were assessed from included studies; study designs, methods for detecting ADEs, techniques for establishing the causal relationship between drug exposures and consequential events, and tools for assessing the preventability and seriousness or severity of ADEs. Out of the ten questions, the total number of "yes" answers was used to compute the quality score for each study. Research studies were assigned a score of ≥ 7 for low risk of bias and ≤ 7 for high risk of bias.

### Data analysis

Studies used different methods to report the occurrence of ADEs; however, we solely extracted prevalence estimates to ensure that the outcome measure was comparable. ADE prevalence was calculated for each study by extracting the total number of patients who experienced at least one ADE as the numerator and the total number of patients in the study population as the denominator. The prevalence of ADEs was described as a percentage of patients with an ADE. We also calculated the medians and interquartile ranges (IQRs) of ADE prevalence across the included studies. Studies were separated into two different at-risk populations, i.e., those targeting general patients (such as medical ward patients, including patients being treated for a specific disease who might have developed ADEs for medication received for something else) and specific patients (studies that either investigated ADEs caused by clinical condition (such as HIV patients) or ADEs caused by exposure to specific drug(s) (such as antibiotics), and therefore, the results, for example, the proportion of ADEs leading to hospital admissions, ADE prevalence during hospitalization, ADE prevalence in the outpatient, and community setting, were reported for these two different at-risk populations, respectively. A similar approach was followed for the seriousness or severity of ADEs, ADE-related fatality, and preventability of ADEs. Since the included studies were heterogeneous, therefore, we did not carry out a meta-analysis.

## Results

### Search results

**Fig. 1:**
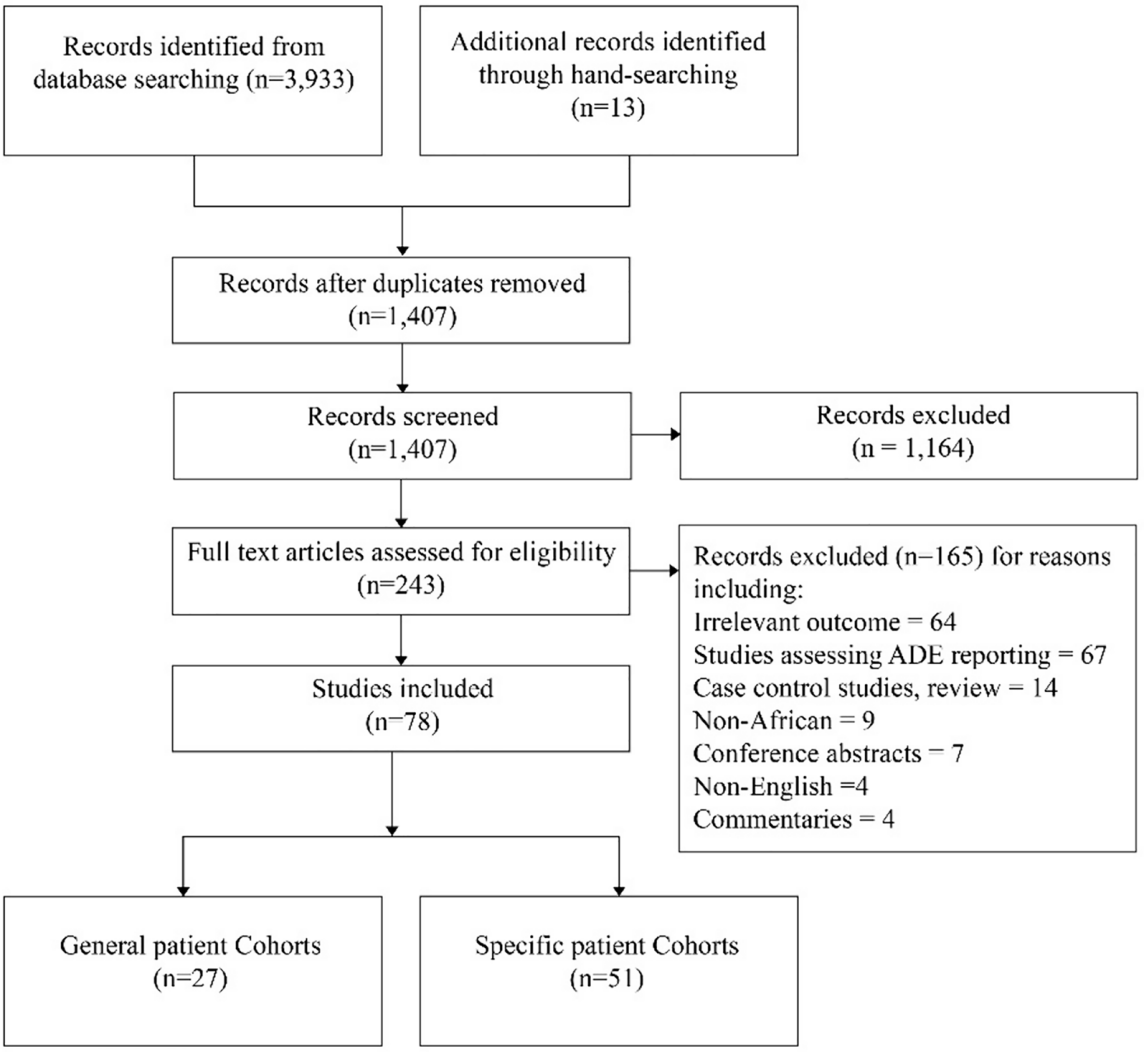
PRISMA flowchart of the study selection process

There were 1407 articles yielded after removing duplicates. Two hundred and forty-three articles qualified for full-text review. A total of 78 articles were found to be suitable for inclusion in the review (Fig. 1). The 78 articles (Tables 1 and 2) included in the analysis were pooled from 15 African countries, i.e., Ethiopia (22), South Africa (11), Nigeria (12), Morocco (4), Cameroon (4), Uganda (10), Mali (2), Kenya (3), Tunisia (1), Ghana (2), Eritrea (2), Malawi (1), Zimbabwe (1), Tanzania (1), and Namibia (2). Detailed characteristics of ADEs studies carried out on general and specific patient Cohorts can be found in Additional file 2: Tables S1 and S2.

**Table 1:** Characteristics of adverse drug events studies carried out on general patient cohorts

Author, Year	Country	Setting
*Adult population*		
Adedapo, 2020 [18]	Nigeria	Medical Wards
Aderemi-Williams Ri, 2015 [19]	Nigeria	Medical Wards
Angamo, 2018 [20]	Ethiopia	Medical Wards
Angamo, 2017 [21]	Ethiopia	Medical Wards
Asio, 2023 [22]	Uganda	Medical and Gynaecological Wards
Ersulo, 2022 [27]	Ethiopia	Medical Ward
Jennane, 2011 [28]	Morocco	ICU
Kiguba, 2017 [29]	Uganda	Medical and Gynaecological Wards
Matsaseng, 2005 [32]	South Africa	Gynaecology Ward
Mehta, 2008 [33]	South Africa	Medical Wards
Mouton, 2016 [34]	South Africa	Medical Wards
Mouton, 2015 [36]	South Africa	Medical Wards
Mouton, 2021 [37]	South Africa	Non-trauma Emergency Unit
Sahilu, 2020 [40]	Ethiopia	Medical Ward
Sendekie, 2023 [41]	Ethiopia	Medical Ward
Tumwikirize, 2011 [43]	Uganda	Medical Wards
*All age groups*		
Benkirane, 2009 [23]	Morocco	ICU
Benkirane, 2009 [24]	Morocco	Medical, Surgical, ICUs, and EDs
Letaief, 2010 [30]	Tunisia	Clinical Departments
*Pediatric population*		
Dedefo, 2016 [25]	Ethiopia	Pediatric Ward
Eshetie, 2015 [26]	Ethiopia	Pediatric Ward
Makiwane, 2019 [31]	South Africa	Pediatric Wards
Mouton, 2020 [35]	South Africa	Medical wards, ICU
Oshikoya, 2011 [38]	Nigeria	Pediatric Ward
Oshikoya, 2007 [39]	Nigeria	Pediatric Ward
*Elderly population*		
Tipping, 2006 [42]	South Africa	Emergency Unit
Yadesa, 2022 [44]	Uganda	Medical, Oncology, and Surgery wards

*ICU* Intensive care unit, *ED* Emergency department

**Table 2:** Characteristics of adverse drug events studies carried out on specific patient cohorts

Author, Year	Country	Setting
*Adult population*		
Abah, 2021 [45]	Nigeria	Outpatient Clinic
Abah, 2018 [46]	Nigeria	Healthcare Facility
Abah, 2015 [47]	Nigeria	HIV Clinic
Abdissa, 2012 [48]	Ethiopia	HIV Outpatient Clinics
Babirye, 2023 [52]	Uganda	Hypertension Clinic
Bahta, 2020 [54]	Eritrea	Outpatient Departments
Berhe, 2017 [55]	Ethiopia	Outpatient Clinics
Beyene, 2022 [56]	Ethiopia	Ambulatory care clinic
Bezabhe, 2015 [57]	Ethiopia	ART Clinics
Chikowe, 2019 [58]	Malawi	Outpatient Department
Elangwe, 2020 [59]	Cameroon	Diabetes Clinic
Elhamdouni, 2020 [60]	Morocco	Multi-centers
Gebremeskel, 2021 [62]	Ethiopia	ART Clinics
Gudina, 2017 [63]	Ethiopia	ART Clinics
Hagos, 2019 [64]	Eritrea	ART Clinic
Kiguba, 2017 [65]	Uganda	Medical and Gynaecological Wards
Kindie, 2017 [67]	Ethiopia	Felege Hiwot Referral Hospital
Luma, 2012 [70]	Cameroon	HIV Outpatient Clinics
Michael, 2016 [72]	Nigeria	Chest Clinic
Mitkie, 2021 [73]	Ethiopia	Multi-Hospitals
Namulindwa, 2022 [74]	Uganda	Immune Suppression Syndrome Clinic
Nemaura, 2013 [76]	Zimbabwe	Outpatient Department
Onoya, 2018 [79]	South Africa	ART Clinics
Otubanjo, 2008 [81]	Nigeria	Communities
Oumar, 2019 [82]	Mali	HIV/AIDS care and Counselling Centre (CESAC)
Sherfa, 2012 [89]	Ethiopia	Public Health Facilities
Tamirat, 2020 [90]	South Ethiopia	ART Clinic
Van Der Walt,2013 [92]	South Africa	Multi-center
Wangai, 2011 [93]	Kenya	HIV Comprehensive Care Clinic
Weldegebreal, 2016 [94]	Ethiopia	ART Unit
*All age groups*		
Amalba, 2021 [50]	Ghana	Chest Clinic
Ategyeka, 2023 [51]	Uganda	TB wards
Eluwa, 2012 [61]	Nigeria	Multi-Hospitals
Kim, 2007 [66]	Kenya,	Outpatient Clinic
Lartey, 2014 [68]	Ghana	Fevers Unit
Isa, 2018 [69]	Nigeria	ART Clinic
Merid, 2019 [71]	Ethiopia	Multi-center
Ndagije, 2018 [75]	Uganda	Health Facilities and Drug Outlets
Njau, 2013 [77]	Tanzania	Health Facilities and Households
Nkenfou-Tchinda, 2020 [78]	Cameroon	Outpatient ART Center
Reginald, 2012 [84]	Nigeria	ART Center
Sagwa, 2014 [85]	Namibia	DR-TB Treatment Ward
Sagwa, 2012 [86]	Namibia	DR-TB Ward
Shean, 2013 [87]	South Africa	XDR-TB Treatment Centers
Shegena, 2022 [88]	Uganda	Medical and Pediatric Ward
*Pediatric population*		
Abdela, 2019 [49]	Ethiopia	ART Clinics
Bahina, 2018 [53]	Cameroon	Community -Home Setting
Opanga, 2019 [80]	Kenya	Pediatric Oncology Ward
Oumar, 2012 [83]	Mali	Pediatric Department
Tola, 2023 [91]	Ethiopia	Pediatric Oncology Unit
Workalemahu, 2020 [95]	Ethiopia	Medical Wards

*TB* Tuberculosis, *ART* Antiretroviral therapy, *DR-TB* Drug resistant tuberculosis, *HIV* Human immune virus, *AIDS* Acquire immune deficiency syndrome, *XDR* Extensively drug resistant, *MDR* Multidrug resistant

### Characteristics of studies

A total of 78 studies investigating ADEs were included in this study (89,899 patients excluding mutual patients from two studies). Study populations were divided into general and specific patient cohorts. Of 78 studies, 27 studies [18–44] were carried out among general patients, while the remaining studies [45–95] were carried out among specific patients. Seventeen studies [18–22, 24, 26, 31, 33–35, 38, 39, 41–43, 51] reported the prevalence of ADEs leading to hospital admissions, while 30 studies [18, 19, 22–33, 35, 37–41, 43, 44, 65, 80, 85–88, 91, 95] reported the prevalence of ADEs during hospitalization. Twelve studies [18, 19, 22, 24, 26, 31, 33, 35, 38, 39, 41, 43] reported ADE occurrence in both settings. In addition, 38 studies [45–50, 52, 54–64, 66–74, 76, 78, 79, 82–84, 89, 90, 92–94] reported the occurrence of ADEs in the outpatient setting, and four studies [53, 75, 77, 81] reported ADEs in the community setting. Majority of included studies [18–22, 27–29, 32–34, 36, 37, 40, 41, 43, 45–48, 52, 54–60, 62–65, 67, 70, 72–74, 76, 79, 81, 82, 89, 90, 92–94] were carried out on adult population (aged ≥ 18 years) (*n* = 46), while 18 studies were conducted among all age groups (aged 0–100 years) [23, 24, 30, 50, 51, 61, 66, 68, 69, 71, 75, 77, 78, 84–88], 12 studies [25, 26, 31, 35, 38, 39, 49, 53, 80, 83, 91, 95] among pediatrics (aged < 18 years) and two studies [42, 44] assessed ADEs in the elderly population (> 65 years).

Thirty-one of the studies were conducted prospectively, while the remaining (*n* = 43) were conducted in a retrospective or cross-sectional manner. Four studies, however, used both retrospective and prospective [39, 63], cross-sectional and analytical study [54] designs and mixed study designs [74]. About 70% of the included studies were carried out at a single healthcare center, while the remaining (30%) were carried out at multi-centers and community settings.

### Quality assessment of studies

All the studies sufficiently described their study design. ADEs were detected in these studies using a variety of approaches, including medical records review as specified by majority of the studies. A variety of ADE confirmation procedures were utilized in the studies, including clinical examination, clinical rounds, patient/caregiver/ward staff interviews, laboratory data review, nursing record review, prescription chart review, and voluntary reporting. Details regarding healthcare professionals engaged in the detection of ADEs were reported in majority of the studies. The process of establishing the causal relationship, preventability, and seriousness or severity were poorly described in the studies. In addition, tools employed in the assessment of causality, preventability, and seriousness or severity were highlighted in 50% of the studies, 28% of the studies, and 53% of the studies, respectively (Table 3). The quality assessment of included studies ranged from 1 to 10 points with a median quality score of 6 (IQR: 4–8) across the 78 studies (Additional file 3: Tables S1 and S2). Thirty-two studies were rated as low risk of bias (scored ≥ 7), while 46 studies were rated as high risk of bias (scored ≤ 7).

**Table 3:** Assessment of methodological quality of included studies (Smyth et al. adapted criteria)

Quality question	Number of “Yes”
*Study design*	
1. Was the study design clear (prospective, retrospective or combined)?	78
*Methods for identifying ADEs*	
2. Were the methods used to identify ADEs described in sufficient detail?	67
3. Were data collection methods (case-record review, drug chart review, and laboratory data) clearly described?	76
4. Were the individuals (clinicians, self-reported, researchers) who identified ADEs clearly described?	67
*Methods for determining causality*	
5. Was the process of establishing the causal relationship described in detail?	28
6. Were standard methods (validated tool) used in the assessment?	39
*Methods for determining PREVETABILITY*	
7. Was the assessment process of establishing PREVETABILITY described in detail?	17
8. Were standard methods (validated tool) used in the assessment?	22
*Methods for determining severity*	
9. Was the assessment process of establishing predictability described in detail?	33
10. Were standard methods (validated tool) used in the assessment?	41

### Studies with a focus on general patient cohorts

There was a total of 27 studies with 22,093 participants involved in the studies (excluding mutual patients from Angamo et al. [20, 21] and Mouton et al. [34, 36] studies). The proportion of females included in the study ranged from 70 to 100%. Six studies [25, 26, 31, 35, 38, 39] were conducted among pediatric patients, two studies [42, 44] on elderly patients, and one study [32] on an all-female population. These 27 studies with a focus on general patient groups were carried out in 6 African countries; Nigeria (4), Morocco (3), Uganda (4), South Africa (8), Tunisia (1), and Ethiopia (7). Twenty-two studies [18–22, 24–33, 35, 38–42, 44] were carried out at a single center, while five studies [23, 34, 36, 37, 43] were conducted at a multi-center. There was a total of 15 studies [18, 21, 23, 25–29, 31, 33, 38, 40–42, 44] that were conducted prospectively, five studies [19, 24, 30, 32, 37] used a retrospective approach; whereas the remaining studies used a cross-sectional study design [20, 22, 34–36, 43]. Oshikoya et al.’s [39] study used both retrospective and prospective study designs. The majority of included studies were carried out among patients admitted to medical wards. The WHO definition for ADRs was frequently used in the included studies [18–22, 29, 31, 33, 39, 43]. The majority of included studies used medical record review [18–22, 25–41, 43, 44] for the detection of ADEs. Twenty-five studies reported on causality assessment, with the Naranjo and WHO-UMC methods frequently referenced. Seriousness or severity of ADEs were assessed in a total of 22 studies, using Temple tools in four studies and other tools in 17 studies. However, the tool employed in the assessment of seriousness or severity of ADEs was not provided in one study [18]. Twenty-three studies conducted an assessment on the preventability of ADEs with Schumock and Thornton criteria employed in the majority of the studies. However, tools employed in ADE preventability assessment were not provided in five studies [18, 23, 25, 31, 39]. We found no studies conducted in the general patient populations reporting the prevalence of ADEs in the outpatient or community settings.

Seventeen studies [18–22, 24, 26, 31, 33–36, 38, 39, 41–43] reported on the prevalence of ADEs leading to hospital admissions ranging from 0.4% to 50% of all study populations; however, data were available for 16 studies. The overall median ADE prevalence among the 16 studies was 6.0% (IQR: 1.5%–9.0%) of all study populations (Table 4).

**Table 4:** Adverse drug event results in general patient cohorts

Study characteristic	Median prevalence (%)	IQR (%)
ADEs leading to hospital Admissions	6.0	5.0–9.0
ADEs During Hospitalization	7.8	4.2–21.4
Seriousness or Severity of ADEs	25.0	7.5–49.0
ADE-Related Fatality	0.1	0.1–0.4
Preventability of ADEs	45.0	30.0–59.0
*Age groups*		
Adults	12.5	9.4–24.9
All age groups	–	–
Pediatrics	7.9	3.2–13.6
Elderly	–	–

*ADEs* Adverse drug events, *IQR* Interquartile range

The prevalence of ADEs during hospitalization was recorded in 22 studies, [18–33, 35, 37–41, 43, 44] with a reported prevalence ranging from 0.6% to 50%. Yadesa et al. [44] reported that 48.9% of elderly patients (> 65 years) experienced ADEs during hospitalization. The overall median prevalence rate among the 22 studies was 7.8% (IQR: 4.2%–21.4%).

Seriousness or severity of ADEs was assessed in a total of 22 studies [18, 20, 22–35, 37–41, 43]; however, data were only available for 19 studies. The assessment of the seriousness or severity of ADEs varied between studies. ADE severity was reported in ten studies [18, 22, 25–27, 37–41], while eight studies [23, 24, 28, 29, 33–35, 43] reported on the seriousness of ADEs. Makiwane et al. [31], however, provided data on both severity and seriousness of ADRs, where the proportion of severe ADRs and serious ADRs were 11·5% and 55·7%, respectively. A South African study [35] focused primarily on serious ADRs, where the prevalence of serious ADRs was considered as their primary outcome measure. They reported that 11·3% of serious ADRs caused admissions, while 8·8% prolonged hospitalizations. Tumwikirize et al. [43] reported that there were no serious ADEs in their study. The reported occurrence of serious or severe ADEs among the studies varied from 1.6% to 87.5%. The overall median proportion of serious or severe ADEs among the 19 studies was 25.0% (IQR: 7.5%–49.0%) of all ADEs. The reported ADE-related fatality rate ranged from 0.1% to 3.2% among 14 studies. Six studies [25, 27, 29, 31, 41, 43] reported no occurrence of ADE-related fatality. Angamo et al. [20] and Mouton et al. [36] focused exclusively on mortality associated with ADEs, investigating the proportion of deaths attributed to ADEs in medical inpatients. Angamo et al. [20] reported that 1·5% of deaths were attributed to ADEs in hospitalized patients while Mouton et al. [36] reported 2·9% and 16·0% of deaths during hospitalizations and all in-hospital deaths were related to ADEs, respectively. The median proportion of fatal ADEs reported for the 14 studies was 0.1% (IQR: 0.1%–0.4%) of all study populations.

Twenty-three studies [18, 20–27, 29–41, 43] carried out preventability assessments and found that the median proportion of preventable ADEs ranged from 4.1% to 97.7% of all ADEs. However, data for two studies [30, 32] were not published, as ADE-specific data could not be recovered. The reported data of all adverse events (i.e., drug and non-drug related) in the two studies revealed that 60.0% and 52.0% of events were preventable, respectively. The median proportion of preventable ADEs reported among the studies was 45.0% (IQR: 30.0%–59.0%) of all ADEs. Elaborated ADE results in general patient cohorts is provided in Additional file 4, Table S4.

The prevalence of ADEs that occurred among the adult population (aged ≥ 18 years) was 12.5% (IQR: 9.4%–24.9%) of all study populations and 7.9% (IQR: 3.2%–13.6%) in pediatric patients. Furthermore, two studies reported that 14.3% and 48.9% of elderly patients (> 65 years) experienced ADEs whilst three studies reported 11.5%, 5.6% and 0.6% prevalence of ADEs among all age groups (aged 0–100 years).

### Studies with a focus on specific patient cohorts

Fifty-one studies were carried out on specific patients, with a total of 67,806 patients. The proportion of females included in this study ranged from 21·0% to 75·9%. These studies were carried out in 14 African countries, including Nigeria (8), Morocco (1), Uganda (6), South Africa (3), Tanzania (1), Ethiopia (15), Ghana (2), Kenya (3), Cameroon (4), Mali (2), Malawi (1), Namibia (2), Zimbabwe (1) and Eritrea (2). Thirty-two studies [45–48, 50, 52, 54, 56, 58, 59, 62, 64–70, 72, 74, 78, 80, 82–86, 88, 90, 91, 93, 94] were carried out at a single center, 15 studies [49, 51, 55, 57, 60, 61, 63, 71, 73, 76, 79, 87, 89, 92, 95] were conducted at a multi-center, and four studies [53, 75, 77, 81] were conducted in the community setting. The community setting comprised home settings, community health facilities, and drug outlets. Sixteen studies [48, 53, 56, 57, 60, 65, 69, 72, 75, 79, 82–84, 88, 91, 92] were conducted prospectively, 20 studies [45–47, 49, 51, 61, 62, 64, 66, 67, 71, 73, 78, 80, 81, 85, 87, 89, 93, 94] were conducted retrospectively, and 12 studies [50, 52, 55, 58, 59, 68, 70, 76, 77, 86, 90, 95] used the cross-sectional study design. Gudina et al. [63] used both retrospective and prospective study designs, Bahta et al. [54] used both cross-sectional and analytical study designs while Namulindwa et al. [74] used mixed design study. The majority of these studies targeted patients with human immunodeficiency virus/acquired immunodeficiency syndrome (HIV/AIDS) [45–49, 57, 61–64, 66–70, 73, 74, 78, 79, 82–84, 89, 90, 93, 94]. Eight studies [50, 51, 60, 71, 85–87, 92] focused on tuberculosis (TB) patients, while Michael et al. [72] and Nemaura et al. [76] targeted patients co-infected with HIV and TB. In addition, three studies [80, 91, 95] focused on pediatric patients (< 15 years) suffering from cancer, while Babirye et al. [52], Berhe et al. [55] and Elangwe et al. [59] investigated ADEs in patients with hypertension and Type II diabetes mellitus, respectively. In addition, three studies [53, 75, 77] focused on malaria patients. Beyene et al. [56] and Shegena et al. [88] targeted epileptic patients and heart failure patients, respectively. A Ugandan study [65] examined antibiotic-associated suspected ADR among hospitalized patients, while a Nigerian study [81] evaluated ADE to ivermectin. Bahta et al. [54] and Chikowe et al. [58] assessed ADEs associated with mental disorders. Seventy-five percent of the studies were carried out in outpatient clinics, while the remaining studies were carried out in medical wards, pediatric oncology wards, DR-TB treatment wards, and community settings. Thirty-one studies were carried out on Adult populations, 14 studies on all age groups while six studies were performed among pediatric patients [49, 53, 80, 83, 91, 95]. The WHO definition of ADRs was frequently used in most studies. The majority of included studies used medical record review for the detection of ADEs. Fourteen studies [52, 57, 59, 60, 64, 65, 69, 75, 82–84, 88, 91, 95] reported causality assessment, with the Naranjo criteria most frequently referenced. An evaluation of the seriousness or severity of ADEs was performed in 31 studies. Twenty studies [45, 47, 57–61, 63–65, 69, 74, 76, 83, 85, 87, 88, 91, 94, 95] highlighted the tools that were employed in the assessment of seriousness or severity of ADEs, with the most common tool used being the WHO criteria. Five studies [57, 65, 75, 83, 87] analyzed the preventability of ADEs, with Schumock criteria employed in four studies. We found one study [51] reporting ADE as a direct cause of admission among specific patient populations. One study [51] reported the prevalence of ADEs leading to hospital admissions, with a reported prevalence of 16.9% of its TB patients (Table 5).

**Table 5:** Adverse drug event results in specific patient cohorts

Study characteristic	Median prevalence (%)	IQR (%)
ADEs leading to hospital Admissions	–	–
ADEs During Hospitalization	74.2	54.1–90.7
ADEs in Outpatient Setting	22.9	14.6–56.1
ADEs in Community Setting	32.6	26.0–41.3
Seriousness or Severity of ADEs	16.2	8.2–32.5
ADE-Related Fatality	1.3	0–7.5
Preventability of ADEs	12.4	7.1–28.1
*Age groups*		
Adults	24.3	17.8–51.5
All age groups	51.2	19.7–62.2
Pediatrics	34.3	18.0–80.1
Elderly	–	–

*ADEs* Adverse drug events, *IQR* Interquartile range

The prevalence of ADEs during hospitalization was recorded in eight studies [65, 80, 85–88, 91, 95] ranging from 19.0% to 100%. A higher ADE prevalence of 92.9% and 100% were recorded in pediatrics cancer patients (< 15 years) [80, 91] and 89.0% and 90.0% in TB patients [85, 86] in four studies, respectively. The ADE occurrence was extraordinarily high in these studies, highlighting priority areas for improvement. The median prevalence of ADEs occurring during hospitalization among the eight studies was 74.2% (IQR: 54.1%–90.7%) of all patients.

Thirty-six studies [45–50, 52, 54–61, 63, 64, 66–74, 76, 78, 79, 82–84, 89, 90, 93, 94] reported data on the prevalence of ADEs in the outpatient setting ranging from 4.3% to 100%. Gebremeskel et al. [62] and Van Der Walt [92] did not provide separate data for ADE prevalence in their study. Five studies reported a higher ADE prevalence of 93.8% and 100% in patients with mental disorders [54, 58], 85.8% [57] in HIV patients, 83.0% in patients co-infected with HIV and TB [76] and 77.3% in TB patients [50], respectively. The overall median prevalence of ADEs among the 36 studies was 22.9% (IQR: 14.6%–56.1%) of all study populations.

Four studies [53, 75, 77, 81] reported ADE prevalence in the community setting ranging from 22.5% to 50.7%. A higher prevalence of 50.7% was reported in malaria patients [77]*.* The overall median ADE prevalence was 32.6% (IQR: 26.0%–41.3%) of all study populations in the community setting. Seriousness or severity of ADEs was assessed in a total of 31 studies; however, data were only reported in 26 studies [45–49, 51, 57–66, 69, 72, 83, 84, 86–88, 91, 92, 94]. The proportion of serious or severe ADEs reported in the 26 studies ranged from 1.2% to 80.3% of all observations. Severe ADEs was reported in 23 studies [45–49, 51, 57–63, 66, 69, 83, 84, 86–88, 91, 92, 94] while three studies [64, 65, 72] reported serious ADEs. Gebremeskel et al. [62] assessed the incidence and predictors of severe ADRs as a primary outcome measure, while Sagwa et al. [85] exclusively reported the occurrence of moderate-severe ADEs occurring during drug-resistant tuberculosis (DR-TB) treatments. Sagwa et al. [85] reported that 51·0% of their study subjects experienced moderate to severe ADEs. Van der Walt et al. [92] exclusively focused on the impact of severe ADR on MDR-TB patients as a primary outcome measure. A higher proportion of 80.3% [94], 57.0% [46], 56.4% [47] of ADEs in HIV patients and 52.2% [49] in pediatric HIV patients (< 15 years) were reported as severe in four studies. The overall median proportion of serious or severe ADEs reported among the 26 studies was 16.2% (IQR: 8.2%–32.5%) of all ADEs.

Data for ADE-related fatality were only available for nine studies [60, 70, 72, 77, 84, 87, 89, 91, 94]. Three studies [60, 70, 72] reported no occurrence of ADE-related fatality. Two studies reported a higher ADE-related fatality rate of 9.0% in TB patients [87] and 8.9% in malaria patients [77]. The median ADE-related fatality rate reported was 1.3% (IQR: 0%–7.5%) of all study subjects with certain clinical conditions.

Data for preventability assessment were provided in four studies [57, 65, 75, 88]. Kiguba et al. [65] reported a higher percentage of preventable ADEs of 63.6% in antibiotic-associated suspected ADR among hospitalized patients. The median proportion of preventable ADEs reported among the four studies was 12.4% (IQR: 7.1%–28.1%) of all ADEs. Elaborated ADE results in specific patient cohorts is provided in Additional file 4, Table S5.

The prevalence of ADEs that occurred among the adult population (aged ≥ 18 years) was 24.3% (IQR: 17.8%–51.5%) of all study populations. We observed a pooled prevalence of 51.2% (IQR: 19.7%–62.2%) among all age groups (aged 0–100 years) and 34.3% (IQR: 18.0%–80.1%) in pediatric patients (aged < 18). We found no studies carried out in specific patient populations that reported the occurrence of ADEs in elderly patients (> 65 years).

## Discussion

To the best of our knowledge, this is the largest systematic review to synthesize existing evidence of ADE prevalence rates and report the nature of ADEs from four different settings in Africa. Our findings confirm the challenges of ADEs on African healthcare systems, and highlight the importance to invest in comprehensive ADE prevention and control including surveillance and monitoring systems, targeted capacity building programs, and efforts towards risk-stratified care, which are vital but unfortunately missing in many African settings. Systematic reviews of ADE occurrence in different settings are scarce in Africa, thereby making a comparison to earlier results difficult. A review by Moulton et al. [10] reviewed the burden of serious ADRs in Sub-Saharan Africa and reported a median proportion of admissions attributed to ADRs was 4.8%, which was comparable to the finding from our study (6.0%). In addition, the prevalence of ADE-related hospital admissions aligned with data from international literature [96, 97]. Tache et al. [96] reviewed the prevalence of ADEs in ambulatory care settings and reported that 5.1% of hospital admissions were due to ADRs, whilst Kongkaew et al. [97] found a median of 5.3% of hospital admissions were caused by ADR.

We found a higher median ADE prevalence during hospitalization (74.2%) in specific patients than the general patients (7.8%). Differences in drug burden may be one of the main causes of this observed disparity, considering that the specific patient cohorts included a greater proportion of cancer, tuberculosis and HIV patients who would require several drugs for continuous therapies and, therefore, at high risk of potential drug-drug interactions leading to ADEs. Notwithstanding, the results as observed for general patients is comparable with results reported by Krähenbühl-Melcher et al. [98] (6.0%) of all hospitalized patients, and Patel et al. [99] (6.3%) during hospitalization. This finding was also similar to a previous systematic review performed by Mekonnen et al. [7] in African hospital setting, where a median estimate for ADEs that occurred during hospitalization was 7.5%.

In the outpatient settings, we discovered a higher median prevalence of ADEs (22.9%), compared to previous studies. An earlier systematic review found that the prevalence of ADEs was 12.8% in the ambulatory setting [96]. Insani et al. [17] reported an ADR prevalence of 8.3% in the primary care setting. This difference might result from different ADE detection methods, as all studies in our review used multiple strategies for ADE detection. In addition, included studies in the outpatient setting involved specific patients (such as HIV/AIDS patients who were vulnerable to ADEs). Outpatient ADEs were likely to be higher due to the capture of patients with a wide range of mild, moderate, and severe symptoms. These factors were also likely to have contributed to the higher ADE prevalence observed in our review*.* We observed extraordinarily high rates of ADE occurrence in some scenario. Given the existence of established countermeasures that demonstrate field effectiveness in risk mitigation for reduction of ADEs [100–103], we recommend ADE prevention and control initiatives aimed at reducing the burden of ADEs in Africa such as nurse and pharmacist-led medication monitoring as well as educational sessions to enhance ADE awareness across various departments in the healthcare systems.

Notwithstanding variations in the seriousness or severity of ADEs reported among the studies, we reported a median proportion of 25.0% and 16.2% of serious or severe ADEs in general and specific patients, respectively, which was in sharp contrast with a much lower ADE fatality rate of 0.1% and 1.3% in our review. This might imply that fatal ADEs were likely to be underreported in studies carried out in Africa. Of 78 included studies, only 28 studies reported data on ADE-related fatality. On the other hand, such rare occurrence of fatal ADEs was consistent with previous studies, as evidenced by Patel et al. [99] (0.08%), indicating improved quality of healthcare in terms of saving lives in African settings. Without any further improvements in ADE prevention and control, this we presume might have an unexpected consequence; in that a shift in healthcare burden might occur from decreasing death tolls to rising hospitalizations as what we observed with respect to the serious or severe ADE occurrence in the current review. This alarming finding suggests continuing efforts should be committed to assure safer medication use for patients in Africa.

The proportion of preventable ADEs was 45.0% and 12.4% in general and specific patient cohorts, respectively. This finding suggests that a higher occurrence of ADEs in general patients are more preventable compared to ADEs that occur in settings where patients are vulnerable and are subjected to complex drug regimens. The under-reported data of preventable ADEs among included studies of specific patient cohorts might be the reason for this observed disparity. The finding in general patients was consistent with two previous reviews [7, 104]. Hakkarainen et al. [104] reported that preventable ADRs among inpatients and outpatients were 52.0% and 45.0%, respectively, while Mekonnen et al. [7] estimated that 43.5% of ADEs were deemed preventable. Adaption of advanced monitoring and reporting systems, as well as appropriate prescribing practices might help improve medication safety in Africa.

ADE prevalence varied across the various age groups, with pediatrics in specific patient cohorts experiencing higher ADE prevalence relative to adults. This is not surprising due to the relatively lesser availability of pediatrics age-tailored medications putting them at a higher risk of under and overdosing. In addition, timely diagnosis of ADEs among pediatrics is challenging due to their difficulties in communicating and describing ADE related symptoms being experienced effectively [105]. The comparison of age-related ADE prevalence with previous studies is challenging, mainly because our studies vary in various aspects, including diverse population characteristics and clinical settings. This may explain the variation in the prevalence rate reported.

The most important strength of this review is the comprehensive assessment of the occurrence and nature of ADEs reported from a large number of the most recent studies in both specific and general patient cohorts in four different settings in Africa. This review also adopted a broader inclusion criterion consisting of studies published on ADEs caused by specific drug (s) or clinical conditions. There are several limitations to be considered. First, there was a limited number of high-quality studies assessing the prevalence of ADEs in African settings. There were considerable variations in the study populations, study length, sample size, and study settings among included studies. Information regarding the detection and assessment of causality, preventability, and seriousness or severity of ADEs were poorly reported. Second, there were also heterogeneities concerning the methods for identifying ADE occurrence and their definitions, which may be one of the reasons for a wide range of estimates across the reviewed studies. Third, ADE prevalence data were not always clearly described in the publications. In addition, prevalence should be derived from whole population. However, the included studies estimated the prevalence rates of ADEs across vastly differing patient groups, which may not be particularly useful as underlying conditions and drug exposure may widely differ. Although we provided the median prevalence rates for general patients (i.e., ADEs occurring in all patients treated within a certain healthcare setting) and specific patient groups (i.e., ADEs caused by clinical condition (such as HIV patients) or ADEs caused by exposure to specific drug(s) (such as antibiotics), results should be interpreted with care. Finally, there were great variations and inconsistencies in the type of drugs in association with observed ADEs, making it a challenge to summarize reports on causative agents.

## Conclusion

ADEs considerably exacerbates the challenges affecting the progress of the healthcare systems in Africa with significant fraction of ADEs being preventable. Our findings indicate a higher ADE prevalence in specific patients and emphasize important areas for improvement, particularly in patients at high risk (e.g., pediatrics, HIV, TB patients) in various settings. This review also demonstrates the challenges of ADEs in different settings in Africa and the severity or seriousness of ADEs, which calls for targeted preventive strategies particularly in high-risk patients to help lessen the burden of ADEs on the healthcare system. There was a limited number of studies concerning ADEs occurring in community settings, pediatric patients and the elderly population; therefore, future studies should focus on this setting and these population groups in Africa.

### Supplementary Information

Additional file 1.

Additional file 2.

Additional file 3.

Additional file 4.

## Data Availability

The authors confirm that data supporting this study are available within the text and its supplementary materials.
