# Supplementary material for: Variation of adverse drug events in different settings in Africa: a systematic review
Source: Eur J Med Res. 2024 Jun 16;29:333. doi: 10.1186/s40001-024-01934-0 (PMC11181533; doi:10.1186/s40001-024-01934-0)
Supplement: Supplementary file 1 — Additional file 1. [file 40001_2024_1934_MOESM1_ESM.pdf]

Additional File 1: Search strategy for PubMed, EBSCO, Science Direct, and Web of Science.

# Step	Search term
1	TI= ‘‘adverse reactions’’ OR ‘‘adverse drug reaction’’ OR ‘‘adverse drug events’’ OR ‘‘adverse events’’ OR ‘‘drug side effects’’ OR ‘‘Toxicity’’.
2	TX= ‘‘hospital’’ OR ‘‘admission’’ OR ‘‘Outpatient’’ OR ‘‘community’’
3	MW=‘‘Morocco’’ OR ‘‘Nigeria’’ OR ‘‘Ethiopia’’ OR ‘‘Egypt’’ OR ‘‘DR Congo’’ OR ‘‘Tanzania’’ OR ‘‘South Africa’’ OR ‘‘Kenya’’ OR ‘‘Uganda’’ OR ‘‘Algeria’’ OR ‘‘Sudan’’ OR ‘‘Morocco’’ OR ‘‘Angola’’ OR ‘‘Mozambique’’ OR ‘‘Ghana’’ OR ‘‘Madagascar’’ OR ‘‘Cameroon’’ OR ‘‘Cote Ivoire’’ OR ‘‘Niger’’ OR ‘‘Burkina Faso’’ OR ‘‘Mali’’ OR ‘‘Malawi’’ OR ‘‘Zambia’’ OR ‘‘Senegal’’ OR ‘‘Chad’’ OR ‘‘Somalia’’ OR ‘‘Zimbabwe’’ OR ‘‘Guinea’’ OR ‘‘Rwanda’’ OR ‘‘Benin’’ OR ‘‘Burundi’’ OR ‘‘Tunisia’’ OR ‘‘South Sudan’’ OR ‘‘Togo’’ OR ‘‘Sierra Leon’’ OR ‘‘Libya’’ OR ‘‘Central Africa Republic’’ OR ‘‘Mauritania’’ OR ‘‘Eritrea’’ OR ‘‘Namibia’’ OR ‘‘Gambia’’ OR ‘‘Botswana’’ OR ‘‘Gabon’’ OR ‘‘Lesotho’’ OR ‘‘Guinea-Bissau’’ OR ‘‘Equatorial Guinea’’ OR ‘‘Mauritius’’ OR ‘‘Eswatini’’ OR ‘‘Djibouti’’ OR ‘‘Comoros’’ OR ‘‘Cabo Verde’’ OR ‘‘Sao Tome and Principe’’ OR ‘‘Seychelles’’.
4	Step 1 AND Step 2 AND Step 3

TI title, *TX* full-text (title, abstract, keywords, and other fields), *MW* subject heading.
