# Supplementary material for: Variation of adverse drug events in different settings in Africa: a systematic review
Source: Eur J Med Res. 2024 Jun 16;29:333. doi: 10.1186/s40001-024-01934-0 (PMC11181533; doi:10.1186/s40001-024-01934-0)
Supplement: Supplementary file 2 — Additional file 2. [file 40001_2024_1934_MOESM2_ESM.pdf]

Additional File 2:

Table S1 Characteristics of Adverse drug events studies carried out on general patient Cohorts

Author, Year	Country	Data Sources & Duration	Study Design	Setting	ADE Definition	Population's Characteristics	Method of Detection	ADE Assessment		
								Causality	Seriousness or severity	Preventability
Adult Population										
Adedapo, 2020 [18]	Nigeria	Single-Center (May 2012 to April 2013)	Prospective Cohort Study	Medical Wards	ADR (WHO)	N= 1280<br>Adult<br>41.8% Female	Examination of medical, and nursing records, reviewing prescription charts, and patient interviews.	WHO-UMC	Assessed but NR	Assessed but NR
Aderemi-Williams Ri, 2015 [19]	Nigeria	Single-Center (January 2009, to December 2009)	Retrospective Chart Review	Medical Wards	ADR (WHO)	N=624<br>Adult<br>42.6% female	Medical record review	NR	NR	NR
Angamo, 2018 [20]	Ethiopia	Single-Center (May 2015 to August 2016)	Cross-Sectional Observational Study	Medical Wards	ADR (WHO)	N= 1001 Adult 45.5% Female	Review of medical records, laboratory tests, and patient interviews.	Naranjo	Hartwig	Schumock
Angamo, 2017 [21]	Ethiopia	Single-Center (May 2015 to August 2016).	Prospective Cross-sectional Study	Medical Wards	ADR (WHO)	N= 1001 Adult 45.5% Female	Review of medical records, laboratory tests, patient interviews, and physical observation.	Naranjo	NR	Schumock
Asio, 2023 [22]	Uganda	Single-Center (November 2013 to April 2014)	Cross-sectional Study	Medical and Gynaecological Wards	ADR(WHO)	N=762<br>Adult<br>70% Female	Clinical charts	Naranjo	DAIDS AE Grading Table	Schumock
Ersulo, 2022 [27]	Ethiopia	Single-Center (February 2021 to July 2021)	Prospective observational study	Medical Ward	ADE (Jha)	N=240<br>Adult<br>52.5% Female	Medical charts, patient interview, Laboratory data	Naranjo	Hartwig	Schumock
Jennane, 2011 [28]	Morocco	Single-Center (during six weeks	Prospective Cohort Study	ICU	ADE (National Coordinating	N=63	Clinical round, voluntary, and verbal report, chart Review, assessing prescriptions and transcriptions voluntary,	NR	WHO	NR

		of September 2009)			Council for Medication Error Reporting and Prevention)	Adult<br>41% female	and verbally report by medical and paramedical staff, chart review and studying prescriptions and transcriptions			
Kiguba, 2017 [29]	Uganda	Single-Center (December 2013 to April 2014)	Prospective Cohort Study	Medical and Gynaecological Wards	ADR (WHO)	N=762<br>Adult<br>70% Female	Clinical Examination, Medical Record Review, Patient/Caregiver/Ward Staff Interviews	Naranjo	DAIDS AE Grading Table	Schumock
Matsaseng, 2005 [32]	South Africa	Single-Center (9-month period)	Retrospective Chart Review	Gynaecology Ward	AE (Brennan)	N=793<br>All Female	Medical record review	Leappe	Brennan	Leappe
Mehta, 2008 [33]	South Africa	Single-Center (September to November 2005)	Prospective Observational Study	Medical Wards	ADR (WHO)	N=665<br>Adult<br>51% Female	Medical record review	WHO	Temple	Schumock
Mouton, 2016 [34]	South Africa	Multicenter<br>(30-day periods during 2013)	Cross-Sectional Survey	Medical Wards	ADR (Aronson and Ferner)	N=1904<br>Adult<br>56% Female	Medical record review, medication history, and Review of laboratory data	WHO-UMC	Temple	Schumock
Mouton, 2015 [36]	South Africa	Multicenter<br>(April to September 2013)	Cross-Sectional	Medical Wards	ADR (Aronson and Ferner)	N=1904<br>Adult<br>56% female	Medical record review, medication history, review of prescriptions, and laboratory data	WHO-UMC	NR	Schumock
Mouton, 2021 [37]	South Africa	Multicenter (December 2014 to November 2015)	Retrospective Study	Non-trauma Emergency Unit	ADR (Aronson and Ferner)	N = 1010<br>Adult<br>57% Female	Folder reviews	WHO-UMC	Temple	Schumock
Sahilu, 2020 [40]	Ethiopia	Single-Center (3-month)	Prospective Observational Study	Medical Ward	ADE (National action plan for adverse drug event prevention)	N=319<br>Adult<br>50.5% Female	Medical charts, patient interviews, and direct observation.	Naranjo	Hartwig	Schumock
Sendekie, 2023 [41]	Ethiopia	Single-Center (May to October 2022)	Prospective Matched Nested Case–Control Study	Medical Ward	NR	N=206<br>Adult<br>35.4% Female	Patient interviews, patient medical record review, laboratory results and direct observation	Naranjo	Hartwig	NR

Tumwikirize, 2011 [43]	Uganda	Multicenter (July to December 2005.)	Longitudinal Observational Study	Medical Wards	ADR(WHO)	N=728<br>Adult<br>56% Female	History and physical examination, medical Record review	Naranjo	Dorman	Schumock
All age groups										
Benkirane, 2009 [23]	Morocco	Multicenter<br>(April to July 2007)	Prospective Cohort Study	ICU	ADE (Bates)	N=696<br>Adult, and Pediatric 45.4% Female	Daily physician rounds, monitoring for medication ordering, and transcribing, solicited reports from health professionals	Begaud	WHO	Consensus agreement
Benkirane, 2009 [24]	Morocco	Single-Center<br>(13 to 17 December 2004)	Retrospective Cross-sectional	Medical, Surgical, ICUs, and EDs	ADE (Bates)	N=1390<br>Adult and Pediatric 40% Female	Solicited information from clinicians	Begaud	WHO	Schumock
Letaief, 2010 [30]	Tunisia	Single-Center (during 2005)	Retrospective Cohort Study	Clinical Departments	AE (Letaief)	N=620<br>General Population 53.4% Female	Medical record review	Wilson	Wilson	Wilson
Pediatric Population										
Dedefo, 2016 [25]	Ethiopia	Single-Center (February to March 2014).	Prospective Observational Study	Pediatric Ward	ADE (American Society of Health-System)	N=233<br>Pediatric<br>36.1% Female	Chart review, ward round, patient/caregiver interview, voluntary report	Naranjo	NCCMERP	Consensus agreement
Eshetie, 2015 [26]	Ethiopia	Single-Center (February to May 2011)	Prospective Observational Study	Pediatric Ward	ADE (Bates)	N=600<br>Pediatric<br>38.2% Female	Chart review, ward round, patient/caregiver interview, voluntary staff report	WHO-UMC	NCCMERP	Schumock
Makiwane, 2019 [31]	South Africa	Single-Center (December 2015 to February 2016)	Prospective Observational Study	Pediatric Wards	ADR (WHO)	N= 282<br>Pediatric<br>NR	Chart Reviews	Naranjo	Hartwig	Assessed but NR
Mouton, 2020 [35]	South Africa	Single-Center (April to May 2015)	Observational Study	Medical<br>wards, ICU	ADR (Aronson and Ferner)	N=1050<br>Pediatric<br>44% Female	Clinical notes, medication prescription charts, and laboratory results	WHO	Temple	Schumock

Oshikoya, 2011 [38]	Nigeria	Single-Center (July 2006 to December 2007)	Prospective Observational Study	Pediatric Ward	ADR (Edwards and Aronson)	N=2004<br><br>Pediatric<br><br>39% Female	Medical and nursing records review, review of prescription charts, attending clinical rounds, reports from healthcare professionals	Jones	Schirm	Schumock
Oshikoya, 2007 [39]	Nigeria	Single-Center (July 2006 to December 2007)	Retrospective Prospective Study	Pediatric Ward	ADR(WHO)	N=3821<br>Pediatric<br><br>42% Female	Medical and nursing record review, prescription chart review	Jones	Martınez-Mir	Assessed but NR
Elderly Population										
Tipping, 2006 [42]	South Africa	Single-Center (February to May 2005).	Prospective Cross-Sectional Study	Emergency Unit	ADE (*South African Medicines Formulary* (6th Edition))	N=517<br><br>Elderly<br>59% Female	Primary physician and/or principal investigator assessment	Nebeker	NR	NR
Yadesa, 2022 [44]	Uganda	Single-Center (November 2020 to May 2021)	Prospective Cohort	Medical, Oncology, and Surgery wards	ADR (Edwards and Aronson)	N=523<br><br>Elderly<br>48.6 % Female	Medical record review, physical assessments and laboratory data	Naranjo	NR	NR

ADE Adverse drug event, *ADR* Adverse drug reaction, *AE* Adverse event, *ICU* Intensive care unit, *ED* Emergency department, *NR* Not recorded, *NS* Not stated, *DAIDS* Division of AIDS, *NCCMER* National Coordinating Council for Medication Error Reporting and Prevention, *WHO* World Health Organization, *WHO-UMC* World Health Organization-Uppsala Monitoring Centre.

Note: Angamo [20] and Angamo [21] used the same groups of patients but different outcome of interest in both studies; Mouton [34] and Mouton [36], used the same groups of patients but different outcome of interest in both studies.

Table S2 Characteristics of Adverse drug events studies carried out on specific patient Cohorts

Author, Year	Country	Data Sources & Duration	Study Design	Setting	ADE Definition	Population's Characteristics	Method of Detection	ADE Assessment		
								Causality	Seriousness or severity	Preventability
Adult Population										
Abah, 2021 [45]	Nigeria	Single-center (January 2004 to December 2015)	Retrospective Cohort Study	Outpatient Clinic	ADR (WHO)	N=13,983<br>HIV-infected Adult 66% Female	Standard clinical evaluation	NR	WHO	NR
Abah, 2018 [46]	Nigeria	Single-center (June 2004 to February 2012)	Retrospective Cohort Study	Healthcare Facility	ADR (WHO)	N=12115<br>HIV-positive Adult 67.3% female	Electronic medical record system	NR	Assessed but NR	NR
Abah,2015 [47]	Nigeria	Single-center (January 2004 to December 2011)	Retrospective Cohort Study	HIV Clinic	ADR (WHO)	N=2920<br>HIV-1 infected Adult<br>22% Female	Standard clinical procedures, spontaneous reports by patients	NR	WHO and Pharmacov igilance definitions	NR
Abdissa, 2012 [48]	Ethiopia	Single-center<br>(October 2008 to December 2009)	Prospective Observational Study	HIV Outpatient Clinics	NR	N=228<br>HIV positive Adult 64% Female	Structured questionnaire	NR	Assessed but NR	NR
Babirye, 2023 [52]	Uganda	Single-Center (November 2021 to January 2022)	cross-sectional	Hypertension Clinic	ADR (WHO)	N=228<br>Adult hypertensive patients<br>75.9% Female	Medical records	Naranjo	NR	NR
Bahta, 2020 [54]	Eritrea	Single-center (August to October 2018)	Cross-sectional Descriptive, and Analytical Study	Outpatient Departments	ADR (WHO)	N=251<br>Adult with schizophrenia 43% Female	Patients self-administered questionnaires	NR	NR	NR
Berhe, 2017 [55]	Ethiopia	Multicenter<br>NS	Cross-sectional Study	Outpatient Clinics	NR	N=925<br>Hypertensive Adult	Interview, medical records	NR	NR	NR

						63% Female.				
Beyene, 2022 [56]	Ethiopia	Single-Center (September 2020 to May 2021)	Prospective observational	Ambulatory care clinic	ADR (Beyene)	N=320<br>Adult epileptic patients<br>45% Female	interviewing and medical records review	NR	NR	NR
Bezabhe, 2015 [57]	Ethiopia	Multicenter (December 2012 to May 2014)	Prospective Cohort Study	ART Clinics	ADR (WHO)	N=211<br>HIV/AIDS Adult<br>60.2% Female	Patients, caregivers' interviews, and review of patients' medical records	Naranjo	AIDS AE (DAIDS AE) grading table	Schumock
Chikowe, 2019 [58]	Malawi	Single-center<br>(4 weeks)	Cross-sectional Observational Study	Outpatient Department	NR	N= 40<br>Adult with psychotic disorders<br>32.5% Female	Structured self-reporting questionnaire	NR	GASS	NR
Elangwe, 2020 [59]	Cameroon	Single-center<br>(June 2018 to June 2019)	Cross-sectional Study	Diabetes Clinic	NR	N=350<br>T2DM Adult<br>58.8% Female	Self-reported by the patients	Naranjo	Modified Hartwig and Siegel's	NR
Elhamdouni, 2020 [60]	Morocco	Multicenter (January 2014 to January 2016)	Multi-centric Observational Prospective Cohort Study	Multi-centers	NR	N=2532<br>TB 15 years or above<br>28% Female	Clinical charts, medical charts	WHO	WHO	NR
Gebremeskel, 2021 [62]	Ethiopia	Single-center (January 2017 to February 2020)	Retrospective Cohort Study	ART Clinics	ADR (WHO)	N=452<br>HIV-positive Adult<br>51.5% female	ART registry logbook and electronic medical record (EMR) system	NR	Assessed but NR	NR
Gudina, 2017 [63]	Ethiopia	Multicenter<br>(September 2009 to December 2013)	Prospective and Retrospective Cohort Study	ART Clinics	ADR (WHO)	N=3921<br>HIV positive Adult<br>61.9% Female	Clinical and/or laboratory data	NR	WHO	NR
Hagos, 2019 [64]	Eritrea.	Single center (September 2005 to December 2016)	Retrospective Study	ART Clinic	ADR (WHO)	N=309<br>HIV Adult<br>64.1% Female	Patients' clinical cards	Naranjo	ICH E2A guideline	NR

Kiguba, 2017 [65]	Uganda	Single-center (December 2013 to April 2014)	Prospective Cohort Study	Medical and Gynaecological Wards	aa-ADR (WHO)	N=762<br>Adult<br>70% Female	Clinical examination	Naranjo	Division of AIDS Table	Schumock
Kindie, 2017 [67]	Ethiopia	Single-center (July 2011 to June 2016)	Retrospective Study	Felege Hiwot Referral Hospital	ADR (WHO)	N=602<br>HIV positive Adult<br>59.5% Female	Patient card	NR	NR	NR
Luma, 2012 [70]	Cameroon	Single-center (between 2003 to 2009)	Cross-sectional Clinical Chart Review	HIV Outpatient Clinics	ADR (Edwards)	N=339<br>Adult HIV patients 60.2% Female	Clinical chart.	NR	NR	NR
Michael, 2016 [72]	Nigeria	Single-center (January 2006 to June 2007)	Prospective observational Study	Chest Clinic	NR	N=103<br>HIV and TB co-infected Adult<br>38.8% female	Clinical examination and Patient interview	NR	Assessed but NR	NR
Mitkie, 2021 [73]	Ethiopia	Multicenter (July 2006 to August 2017)	Retrospective Cohort	Multi-Hospitals	ADR (Gudina)	N= 592<br>HIV positive Adult<br>65.5% female	Follow-up forms	NR	NR	NR
Namulindwa, 2022 [74]	Uganda	Single-Center	Mixed Design Study	Immune Suppression Syndrome Clinic	NR	N=375<br>Adult HIV patients<br>40.5% Female	Medical files and Patients interview	NR	DAIDS grading of ADEs	NR
Nemaura, 2013 [76]	Zimbabwe	Multicenter<br>(NS)	Cross-sectional, Case-control Study	Outpatient Department	NR	N=430<br>HIV/AIDS and/or TB-infected Adult 65% female	Questionnaire	NR	WHO	NR
Onoya, 2018 [79]	South Africa	Multicenter<br>(April 2004 to January 2016)	Prospective Cohort Study	ART Clinics	NS	N = 7708<br>HIV-1-infected Adult<br>65.0% female	Clinical notes, laboratory data	NR	NR	NR

Otubanjo, 2008 [81]	Nigeria	Communities (1996-2000)	Retrospective Study	Communities	NS	N=2398<br>Adult<br>50.1% Female	Interview	NR	NR	NR
Oumar, 2019 [82]	Mali	Single-center<br>(June 2011 to May 2012)	Prospective Cohort Study	HIV/AIDS care and Counselling Centre (CESAC)	NS	N=843<br>HIV-infected Adult 71.9% Female	Patient interviews, medical charts	Naranjo	NR	NR
Sherfa, 2012 [89]	Ethiopia	Multicenter (January 2013 to December 2018)	Retrospective Cohort Study	Public Health Facilities	ADR (WHO)	N=456<br>HIV Adult 61.18% Female	ART registers and medical record review	NR	NR	NR
Tamirat, 2020 [90]	South Ethiopia	Single-center (2005 to 2014)	Cross-sectional Study	ART Clinic	ADR (WHO)	N= 231<br>HIV/AIDS Adult<br>64.5% Female	Medical records review	NR	Assessed but NR	NR
Van Der Walt,<br>2013 [92]	South Africa	Multicenter<br>(2000 to 2004)	Prospective Cohort	Multi-center	ADR (Van der walt)	N=1390<br>MDR-TB Adult<br>38.9% Female	Patient self-reporting or through observation by the clinician/nurse	NR	Assessed but NR	NR
Wangai, 2011 [93]	Kenya	Single-center (mid-2003 to the end of 2007)	Retrospective Cohort Study Design	HIV Comprehensive Care Clinic	NS	N=413<br>HIV infected Adult 65.9% Female	Medical record	NR	NR	NR
Weldegebreal,<br>2016 [94]	Ethiopia	Single-center (March 2006 to March 2014)	Retrospective study	ART Unit	ADR (Teklay)	N=358<br>HIV/AIDS Adult 68.4% Female	Clinical records	NR	WHO	NR
All age groups										
Amalba, 2021 [50]	Ghana	Single center<br>(October 2017 to April 2018)	Cross-sectional Study	Chest Clinic	ADR (Edwards)	N= 66<br>All patients on first-line anti-tubercular therapy<br>50% Female	Questionnaire	NR	NR	NR

Ategyeka, 2023 [51]	Uganda	Multi-Center (Jan, 2016 to Dec, 2020)	Retrospective cohort	TB wards	NR	N=856<br><br>All MDR-TB patients<br><br>36.6% female	Medical records review	NR	NR	NR
Eluwa, 2012 [61]	Nigeria	Multicenter<br><br>(May 2006 to May 2009)	Retrospective Cohort Analysis	Multi-Hospitals	ADR (WHO)	N=2650<br><br>All patients on ART 64% Female	ADR screening form	NR	WHO	NR
Kim, 2007 [66]	Kenya,	Single center (February 2003 to February 2005)	Retrospective Cohort Study	Outpatient Clinic	NR	N=283<br><br>All HIV patients 70% female	History, clinical examination, and Laboratory test	NR	Assessed but NR	NR
Lartey, 2014 [68]	Ghana	Single-center (December 2003 to June 2007)	Cross-sectional Retrospective study	Fevers Unit	ADR (WHO)	N=167<br><br>All HIV/AIDS patients<br><br>62.3% Female	Review of patient data	NR	NR	NR
Isa, 2018 [69]	Nigeria	Single-center (February to July 2017)	Prospective Observational Study	ART Clinic	ADR (WHO)	N=167<br><br>HIV/AIDS patients > 13 years 60% Female	Patient interviews, medical records, and follow-up	WHO	Hartwig	NR
Merid, 2019 [71]	Ethiopia	Multicenter (September 2010 to December 2017)	Retrospective Cohort Study	Multi-center	NR	N=570<br><br>All DR-TB patients 43.2% Female	Patient charts, medical notes, follow up green cards and registries	NR	NR	NR
Ndagije, 2018 [75]	Uganda	Community (April to July 2017)	Observational, Active, Follow-up Study	Health Facilities and Drug Outlets	ADR (Basu)	N=782<br><br>All Patients treated for uncomplicated malaria<br><br>58.6% Female	Reported by patients, or recorded by healthcare workers	Naranjo	NR	P-method
Njau, 2013 [77]	Tanzania	Rural districts (2003 to 2005).	Cross-sectional Study	Health Facilities and Households	ADR (Phillips-Howard)	N=67<br><br>All patients treated with Sulphadoxine-pyrimethamine and artemsinin	Passive and active surveillance	NR	NR	NR

						60% Female				
Nkenfou-Tchinda, 2020 [78]	Cameroon	Single-center (January 2013 to December 2013)	Retrospective Study	Outpatient ART Center	ADR(Ejigu)	N = 1254<br>All HIV-positive patients<br>female 67.4% Female	Patient complaints and/or observations made and reported in the patient's record	NR	NR	NR
Reginald, 2012 [84]	Nigeria	Single-center<br>(NS)	Prospective Cohort Study	ART Center	ADR (Edwards)	N=3641<br>All HIV- infected patients<br>67.5% female	Reported by patients, or were detected by nurses and clinicians	Naranjo	Assessed but NR	NR
Sagwa, 2014 [85]	Namibia	Single-center (January 2008 to February 2010)	Retrospective Observational Cohort Study	DR-TB Treatment Ward	NS	N=57<br>All Patients with DR-TB<br>NS	Observed by clinician or reported by patient	NR	DR-TB patient treatment booklet	NR
Sagwa, 2012 [86]	Namibia	Single-center (January 2008 to February 2010)	Cross-sectional Descriptive Study	DR-TB Ward	NS	N= 59<br>All Patients with DR-TB<br>34% Female	Patient records	NR	Assessed but NR	NR
Shean, 2013 [87]	South Africa	Multicenter (August 2002 to February 2008)	Retrospective Observational Study	XDR-TB Treatment Centers	NS	N=115<br>All XDR-TB patients<br>NS	Case records	NR	Modified American National Institute of Health common terminology criteria for AE	NR
Shegena, 2022 [88]	Uganda	Single-Center (November 2021 to January 2022)	Prospective Observational Study	Medical and Pediatric Ward	ADR (Edwards and Aronson)	N=118<br>All heart Failure patients<br>61% Female	Medical file and Physical examination	Naranjo	Hartwig	Schumock

Pediatric Population

Abdela, 2019 [49]	Ethiopia	Multicenter (March 2007 to April 2015)	Retrospective Cross-sectional Study	ART Clinics	ADR (WHO)	N=153<br>Pediatric on ART<br>53.6% female	Medical record	NR	Assessed but NR	NR
Bahina, 2018 [53]	Cameroon	Community (January 2013 to November 2013)	Prospective Observational Cohort Study	Community - Home Setting	NR	N=479<br>Pediatrics with malaria<br>43.2% female	An elaborated Pharmacovigilant form Clinical records	NR	NR	NR
Opanga, 2019 [80]	Kenya	Single-center (December 2016 to May 2017)	Observational Study	Pediatric Oncology Ward	NS	N= 85<br>Pediatrics with non-Hodgkin's Lymphomas<br>21% Female	Review of patients' records, retrospective review of patients' records	NR	NR	NR
Oumar, 2012 [83]	Mali	Single-center (June to November 2010)	Prospective Study	Pediatric Department	NS	N= 92<br>HIV-positive pediatric patients<br>44.6% Female	Interview with the patient and/ or the patient's attendants, as well as a review of outpatient case records, laboratory reports, clinicians' notes, and prescriptions at each follow-up visit.	WHO	Modified Hartwig	Modified Shumock
Tola, 2023 [91]	Ethiopia	Single-Center (October and December 2020	Prospective Observational Study	Pediatric Oncology Unit	ADE(Common terminology criteria for adverse<br>events (CTCAE) version 4.0.	N=73<br>Pediatric cancer Patients<br>37% Female	Clinical rounds, Interview children and/or parents/ caregiver and Pediatrics oncology staff voluntary reports.	WHO-UMC	NCI CTCAE	NR
Workalemahu<br>2020 [95]	Ethiopia	Multicenter (July 2017 to August 2019)	Cross-sectional study	Medical Wards	ADR (WHO)	N= 287<br>Pediatric with cancer<br>38.7% Female	Patient charts	WHO-UMC	Hartwig and Siegel	NR

ADE Adverse drug event, *ADR* Adverse drug reaction, *AE* Adverse event, *TB* Tuberculosis, *ART* Antiretroviral therapy, *DR-TB* Drug resistant tuberculosis, *HIV* Human immune virus, *AIDS* Acquire immune deficiency syndrome, *XDR* Extensively drug resistant, *MDR* Multidrug resistant, *NR* Not recorded, *NS* Not stated, *DAIDS* Division of AIDS, *WHO* World Health Organization, *WHO-UMC* World Health Organization-Uppsala Monitoring Centre, *GASS* Glasgow antipsychotic side effects. NCI CTCAE, national cancer institute common terminology criteria for adverse events version.
