# Supplementary material for: Variation of adverse drug events in different settings in Africa: a systematic review
Source: Eur J Med Res. 2024 Jun 16;29:333. doi: 10.1186/s40001-024-01934-0 (PMC11181533; doi:10.1186/s40001-024-01934-0)
Supplement: Supplementary file 3 — Additional file 3. [file 40001_2024_1934_MOESM3_ESM.pdf]

Additional File 3:

Table S1: Overview of methodological quality assessment of included studies in general patient Cohorts

Author, year	1	2	3	4	5	6	7	8	9	10	SUM
Adedapo, 2020 [18]	Y	Y	Y	Y	N	Y	Y	NR	Y	NR	7
Aderemi-Williams Ri, 2015 [19]	Y	NR	Y	NR	N	N	N	N	N	N	2
Angamo,2018[20]	Y	Y	Y	Y	Y	Y	Y	Y	N	Y	9
Angamo, 2017 [21]	Y	Y	Y	Y	Y	Y	Y	Y	N	N	8
Asio, 2023 [22]	Y	Y	Y	Y	Y	Y	NR	Y	NR	Y	8
Benkirane, 2009 [23]	Y	Y	Y	Y	NR	Y	N	NR	Y	Y	7
Benkirane, 2009 [24]	Y	Y	Y	Y	Y	Y	Y	Y	Y	Y	10
Dedefo, 2016 [25]	Y	Y	Y	Y	NR	Y	Y	NR	Y	Y	9
Eshetie, 2015 [26]	Y	Y	Y	Y	Y	Y	Y	Y	Y	Y	10
Ersulo, 2022 [27]	Y	Y	Y	Y	Y	Y	Y	Y	Y	Y	10
Jennane, 2011[28]	Y	Y	Y	Y	N	N	N	N	Y	Y	6
Kiguba, 2017 [29]	Y	Y	Y	Y	Y	Y	Y	Y	Y	Y	10
Letaief, 2010 [30]	Y	N	Y	Y	Y	Y	N	Y	N	Y	7
Makiwane, 2019 [31]	Y	Y	Y	Y	Y	Y	NR	NR	Y	Y	8
Matsaseng, 2005 [32]	Y	NR	Y	Y	Y	Y	Y	Y	NR	Y	8
Mehta, 2008 [33]	Y	Y	Y	Y	Y	Y	Y	Y	Y	Y	10
Mouton, 2016 [34]	Y	Y	Y	Y	Y	Y	Y	Y	Y	Y	10
Mouton, 2020 [35]	Y	Y	Y	Y	Y	Y	Y	Y	Y	Y	10
Mouton, 2015 [36]	Y	Y	Y	Y	Y	Y	Y	Y	N	N	8
Mouton, 2021 [37]	Y	Y	Y	Y	NR	Y	Y	Y	Y	Y	9
Oshikoya, 2011 [38]	Y	Y	Y	Y	Y	Y	Y	Y	Y	Y	10
Oshikoya, 2007 [39]	Y	Y	Y	Y	Y	Y	NR	NR	NR	Y	7
Sahilu, 2020 [40]	Y	Y	Y	Y	Y	Y	N	Y	Y	Y	9
Sendekie, 2023 [41]	Y	Y	Y	Y	Y	Y	N	N	Y	Y	8
Tipping, 2006 [42]	Y	Y	N	Y	NR	Y	N	N	N	N	4
Tumwikirize, 2011 [43]	Y	Y	Y	Y	Y	Y	NR	Y	NR	Y	8
Yadesa, 2022 [44]	Y	Y	Y	Y	Y	Y	N	N	N	N	6

Y Yes, *N* No, *NR* Not reported.

Table S2: Overview of methodological quality assessment of included studies in specific patient Cohorts

Author, year	1	2	3	4	5	6	7	8	9	10	SUM
Abah, 2021[45]	Y	Y	Y	Y	N	N	N	N	Y	Y	6
Abah, 2018 [46]	Y	NR	Y	NR	N	N	N	N	N	N	2
Abah,2015 [47]	Y	Y	Y	NR	N	N	N	N	Y	Y	5
Abdissa, 2012 [48]	Y	Y	Y	Y	N	N	N	N	N	NR	4
Abdela, 2019 [49]	Y	Y	Y	NR	N	N	N	N	N	N	3
Amalba, 2021 [50]	Y	Y	Y	Y	N	N	N	N	N	N	4
Ategyeka, 2023 [51]	Y	Y	Y	N	N	N	N	N	NR	NR	3
Babirye, 2023 [52]	Y	Y	Y	Y	Y	Y	N	N	N	N	6
Bahina, 2018 [53]	Y	NR	Y	Y	N	N	N	N	N	N	3
Bahta, 2020 [54]	Y	Y	Y	Y	N	N	N	N	N	N	4
Berhe, 2017 [55]	Y	Y	Y	Y	N	N	N	N	N	N	4

Beyene, 2022 [56]	Y	Y	Y	Y	N	N	N	N	N	N	4
Bezabhe, 2015 [57]	Y	Y	Y	Y	NR	Y	NR	Y	NR	Y	7
Chikowe, 2019 [58]	Y	NR	Y	NR	N	N	N	N	Y	Y	4
Elangwe, 2020 [59]	Y	Y	Y	Y	NR	Y	N	N	NR	Y	6
Elhamdouni, 2020 [60]	Y	Y	Y	Y	Y	Y	N	N	Y	Y	8
Eluwa, 2012 [61]	Y	Y	Y	Y	N	N	N	N	Y	Y	6
Gebremeskel, 2021 [62]	Y	Y	Y	Y	N	N	N	N	N	N	4
Gudina, 2017 [63]	Y	Y	Y	Y	Y	N	N	N	Y	Y	7
Hagos, 2019 [64]	Y	Y	Y	Y	Y	Y	N	N	Y	Y	8
Kiguba, 2017 [65]	Y	Y	Y	Y	Y	Y	Y	Y	Y	Y	10
Kim, 2007 [66]	Y	Y	Y	Y	N	N	N	N	Y	NR	5
Kindie, 2017 [67]	Y	Y	Y	Y	N	N	N	N	N	N	4
Lartey, 2014 [68]	Y	NR	Y	Y	N	N	N	N	N	N	3
Isa, 2018 [69]	Y	Y	Y	Y	Y	Y	N	N	NR	Y	7
Luma, 2012 [70]	Y	Y	Y	Y	N	N	N	N	N	N	4
Merid, 2019 [71]	Y	NR	Y	Y	N	N	N	N	N	N	3
Michael, 2016 [72]	Y	Y	Y	Y	N	N	N	N	Y	NR	5
Mitkie, 2021[73]	Y	NR	Y	NR	N	N	N	N	N	N	2
Namulindwa, 2022 [74]	Y	Y	Y	Y	N	N	N	N	Y	Y	6
Ndagije, 2018 [75]	Y	Y	Y	Y	N	Y	Y	Y	N	N	7
Nemaura, 2013 [76]	Y	Y	Y	Y	N	N	N	N	Y	Y	6
Njau, 2013 [77]	Y	Y	Y	Y	N	N	N	N	N	N	4
Nkenfou-Tchinda, 2020 [78]	Y	Y	Y	Y	N	N	N	N	N	N	4
Onoya, 2018 [79]	Y	Y	Y	NR	N	N	N	N	N	N	3
Opanga, 2019 [80]	Y	Y	Y	NR	N	N	N	N	N	N	3
Otubanjo, 2008 [81]	Y	N	N	N	N	N	N	N	N	N	1
Oumar, 2019 [82]	Y	Y	Y	Y	NR	Y	N	N	N	N	5
Oumar, 2012 [83]	Y	Y	Y	Y	NR	Y	NR	Y	Y	Y	8
Reginald, 2012 [84]	Y	Y	Y	Y	Y	Y	N	N	Y	NR	7
Sagwa, 2014 [85]	Y	Y	Y	Y	N	N	N	N	Y	Y	6
Sagwa, 2012 [86]	Y	NR	Y	Y	N	N	N	N	N	N	3
Shean, 2013 [87]	Y	Y	Y	Y	N	N	N	N	Y	Y	6
Shegena, 2022 [88]	Y	Y	Y	Y	Y	Y	NR	Y	NR	Y	8
Sherfa, 2012 [89]	Y	Y	Y	Y	N	N	N	N	N	N	4
Tamirat, 2020 [90]	Y	Y	Y	Y	N	N	N	N	Y	NR	5
Tola, 2023 [91]	Y	Y	Y	Y	NR	Y	N	N	NR	Y	6
Van Der Walt, 2013 [92]	Y	Y	Y	Y	N	N	N	N	N	N	4
Wangai, 2011 [93]	Y	Y	Y	NR	N	N	N	N	N	N	3
Weldegebreal, 2016 [94]	Y	Y	Y	Y	N	N	N	N	NR	Y	5
Workalemahu 2020 [95]	Y	Y	Y	Y	NR	Y	N	N	NR	Y	6

Y Yes, *N* No, *NR* Not reported.
