# Supplementary material for: Variation of adverse drug events in different settings in Africa: a systematic review
Source: Eur J Med Res. 2024 Jun 16;29:333. doi: 10.1186/s40001-024-01934-0 (PMC11181533; doi:10.1186/s40001-024-01934-0)
Supplement: Supplementary file 4 — Additional file 4. [file 40001_2024_1934_MOESM4_ESM.pdf]

Additional File 4:

Table S4 Adverse drug event results in General Patient Cohorts

Author, year	Prevalence of ADEs leading to hospital Admissions (%)[b]	Prevalence of ADEs during Hospitalization (%)[b]	Seriousness or Severity of ADEs (%)[c]	ADE-Related Fatality (%)[b]	Preventability of ADEs (%)[c]
Adedapo, 2020 [18]	3.9	1.3	65.7	0.5	62.7
Aderemi-Williams Ri, 2015 [19]	6.4	4.3			
Angamo, 2018 [20]	10.3		NS	1.5	93.8
Angamo, 2017 [21]	10.3				89.1
Asio, 2023 [22]	7	6.8[a]	24.7	0.3	46.8
Benkirane, 2009 [23]		11.5	51.8	0.3	30.0
Benkirane, 2009 [24]	1.4	4.2	47.5	0.1	13.2
Dedefo, 2016 [25]		7.3	5.9	0	47.1
Eshetie, 2015 [26]	0.7[a]	7.7[a]	9.0	0.2	33.0
Ersulo, 2022 [27]		24.2	1.6	0	59
Jennane, 2011 [28]		12.7	87.5	3.2	
Kiguba, 2017 [29]		25.5	43.9	0	54.7
Letaief, 2010 [30]		0.6	NS	NS	NS
Makiwane, 2019 [31]	5.7	12.8	55.7	0	38.0
Matsaseng, 2005 [32]		0.59	NS	NS	NS
Mehta, 2008 [33]	6.3	6.2	50.4	0.3	46.0
Mouton, 2016 [34]	8.5[a]		23.2	1.5	45.0
Mouton, 2020 [35]	4.4[a]	10.9[a]	25.0	0.1[a]	15.6
Mouton, 2015 [36]				2.9	43.5
Mouton, 2021 [37]		7.9	2.4[a]	0.1[a]	34.5[a]
Oshikoya, 2011 [38]	0.6	1.2	37.5	0.1	20.0
Oshikoya, 2007 [39]	0.4	0.7	37.5	0.1	97.7
Sahilu, 2020 [40]		26.6	10.3		62.1
Sendekie, 2023 [41]	50	50	1.6	0	6.3
Tipping, 2006 [42]	14.3[a]				
Tumwikirize, 2011 [43]	1.5	49.5	0	0	4.1
Yadesa, 2022 [44]		48.9			
Median (IQR)	6.0(1.5–9.0)	7.8(4.2–21.4)	25.0(7.5–49)	0.1(0.1–0.4)	45.0(30–59.0)

IQR Interquartile Range, *NS* No Specific data available.

[a] Data was not present in the study, but calculated from other data presented in the study; [b] The total number of patients was used as a denominator; [c] The total number of reported ADEs was used as a denominator.

Table S5 Adverse drug event results in Specific Patient Cohorts

Author, year	Prevalence of ADEs leading to Hospital Admissions (%)[b]	Prevalence of ADEs during Hospitalization (%)[b]	Prevalence of ADEs in Outpatient Setting (%)[b]	Prevalence of ADEs In Community Setting (%)[b]	Seriousness or Severity of ADEs (%)[c]	ADE-Related Fatality (%)[b]	Preventability of ADEs (%)[c]
Abah, 2021 [45]			10.6[a]		15.6		
Abah, 2018 [46]			7.9		57.0		
Abah,2015 [47]			12.3[a]		56.4		
Abdissa, 2012 [48]			50.9		7.7[a]		
Abdela, 2019 [49]			15.0		52.2[a]		
Amalba, 2021 [50]			77.3				
Ategyeka, 2023 [51]	16.9				10.5[a]		
Babirye, 2023 [52]			21.1				
Bahina, 2018 [53]				27.1			
Bahta, 2020 [54]			93.8				
Berhe, 2017 [55]			20.9				
Beyene, 2022 [56]			34.4				
Bezabhe, 2015 [57]			85.8		22.1		16.3
Chikowe, 2019 [58]			100		7.5		
Elangwe, 2020 [59]			25.7		19.4		
Elhamdouni, 2020 [60]			10.0		38.7	0	
Eluwa, 2012 [61]			4.3		29		
Gebremeskel, 2021 [62]					33.4		
Gudina, 2017 [63]			22.1		43.3		
Hagos, 2019 [64]			62.8		29.8		
Kiguba, 2017 [65]		19.0			14.0		63.6
Kim, 2007 [66]			65.0		9.8[a]		
Kindie, 2017 [67]			10.1				
Lartey, 2014 [68]			9.4				
Isa, 2018 [69]			58.7		1.2		
Luma, 2012 [70]			19.5			0	
Merid, 2019 [71]			51.2				
Michael, 2016 [72]			53.4		14.5	0	
Mitkie, 2021 [73]			17.9				
Namulindwa, 2022 [74]			33. 1		NS		
Ndagije, 2018 [75]				22.5			3
Nemaura, 2013 [76]			83.0		NS		
Njau, 2013 [77]				50.7		8.9	
Nkenfou-Tchinda, 2020 [78]			24.4				

Onoya, 2018 [79]			44.5				
Opanga, 2019 [80]		92.9					
Otubanjo, 2008 [81]				38.1			
Oumar, 2019 [82]			42.3				
Oumar, 2012 [83]			14.1		7.7	NS	NS
Reginald, 2012 [84]			10.4		16.8	0.1[a]	
Sagwa, 2014 [85]		89.0			NS		
Sagwa, 2012 [86]		90.0			15.0		
Shean, 2013 [87]		58.3			3.0	9.0	
Shegena, 2022 [88]		59.3			7.3		8.5
Sherfa, 2012 [89]			17.3			1.3	
Tamirat, 2020 [90]			22.9		NS		
Tola, 2023 [91]		100			24	6.8	
Van Der Walt, 2013 [92]					6.0		
Wangai, 2011 [93]			63.2				
Weldegebreal, 2016 [94]			17.0		80.3	7.5	
Workalemahu 2020 [95]		41.5			NS		
Median (IQR)	–	74.2(54.1–90.7)	22.9(14.6–56.1)	32.6(26.0–41.3)	16.2(8.2–32.5)	1.3(0–7.5)	12.4(7.1–28.1)

IQR Interquartile Range, *NS* No Specific data Available.

[a]Data was not present in the study, but calculated from other data presented in the study; [b]The total number of patients was used as a denominator; [c] The total number of reported ADEs was used as a denominator.
